# COVID‐19 and neurodegeneration: The mitochondrial connection

**DOI:** 10.1111/acel.13727

**Published:** 2022-10-11

**Authors:** Christopher A. Denaro, Yara I. Haloush, Samuel Y. Hsiao, John J. Orgera, Teresa Osorio, Lindsey M. Riggs, Joshua W. Sassaman, Sarah A. Williams, Anthony R. Monte Carlo, Renata T. Da Costa, Andrey Grigoriev, Maria E. Solesio

**Affiliations:** ^1^ Department of Biology and Center for Computational and Integrative Biology Rutgers University Camden New Jersey USA

**Keywords:** Alzheimer's disease, bioenergetics, COVID‐19, inflammation, mitochondria, neurodegeneration, Parkinson's disease, SARS‐CoV‐2

## Abstract

There is still a significant lack of knowledge regarding many aspects of the etiopathology and consequences of the severe acute respiratory syndrome coronavirus 2 (SARS‐CoV‐2) infection in humans. For example, the variety of molecular mechanisms mediating this infection, and the long‐term consequences of the disease remain poorly understood. It first seemed like the SARS‐CoV‐2 infection primarily caused a serious respiratory syndrome. However, over the last years, an increasing number of studies also pointed towards the damaging effects of this infection has on the central nervous system (CNS). In fact, evidence suggests a possible disruption of the blood–brain barrier and deleterious effects on the CNS, especially in patients who already suffer from other pathologies, such as neurodegenerative disorders. The molecular mechanisms behind these effects on the CNS could involve the dysregulation of mitochondrial physiology, a well‐known early marker of neurodegeneration and a hallmark of aging. Moreover, mitochondria are involved in the activation of the inflammatory response, which has also been broadly described in the CNS in COVID‐19. Here, we critically review the current bibliography regarding the presence of neurodegenerative symptoms in COVID‐19 patients, with a special emphasis on the mitochondrial mechanisms of these disorders.

The severe acute respiratory syndrome coronavirus 2 (SARS‐CoV‐2) was first reported in 2020 (Zhou et al., 2020). SARS‐CoV‐2 infection is the primary cause of the coronavirus disease 2019 (COVID‐19) pandemic (Zhu et al., 2020). The SARS‐CoV‐2 virus is a large, enveloped RNA virus (V'Kovski et al., 2021) with a ~30 Kb genome composed of two major segments. The open reading frame 1ab (ORF1ab) of ~20 Kb contains non‐structural proteins (Nsps), and a 10 Kb segment downstream of ORF1ab encodes structural proteins such as the nucleocapsid (N), spike (S), and membrane (M) proteins (Astuti & Ysrafil., 2020), and protein products of several other ORFs. The canonical mode of SARS‐CoV‐2 infection involves the spike protein attaching to the ACE2 receptor in the host cell membrane. While ACE2 was typically found in the heart, kidney, and gastrointestinal system; a recent study showed ACE2 activity in human neurons and cerebrospinal fluid (Xu & Lazartigues, 2022), as well as in areas of the brain involved in cardiorespiratory function, including the hypothalamus and the brainstem (Netland et al., 2008). The invasion of the host cell begins when the spike is cleaved into two major components, S1 and S2, by the furin protease and type II transmembrane serine protease. S2 then enables the fusion of the virus and cell membranes, the virus genome enters the host cell and viral proteins are translated from the Plus‐strand RNA and subgenomic mRNAs, produced later (Astuti & Ysrafil., 2020). Other modes of infection (e.g., involving cell–cell fusion leading to the formation of syncythia (Braga et al., 2021)) have also been described.

When the first cases of COVID‐19 were reported, the prevailing view confined the most severe clinical disease outcomes to the respiratory system. However, recent work has shown the neuroinvasive potential of SARS‐CoV‐2. Neurological alterations have been reported already in the acute phase of COVID‐19, and in some cases as long‐term sequelae (Filatov et al., 2020). Increased rate of cell death within the central nervous system (CNS) has been observed as the infection progressed (Song et al., 2021). Further, it has been reported that the invasion of neural cells by SARS‐CoV‐2 could disrupt the stability of the blood–brain barrier (BBB) (Krasemann, Haferkamp, et al., 2022b; Rhea et al., 2021; Song et al., 2021; Zhang et al., 2021). This may allow either the virus or some viral proteins/their fragments to cross the BBB to reach microglia cells (Jeong et al., 2022) (via a mechanism that is not yet totally understood, but possibly involving trans‐cellular passage (Zhang et al., 2021)). While some authors support that SARS‐CoV‐2 physically crosses the BBB (Hansen et al., 2018; Jeong et al., 2022; Mazza et al., 2021; Netland et al., 2008; Xu & Lazartigues, 2022; Zhou et al., 2021), this still remains controversial. Detection of the virus even in the most detailed studies (Krasemann, Haferkamp, et al., 2022b; Matschke et al., 2020) is based on PCR amplification of short sections of genomic RNA and on some of the proteins of the virus, but not supported at the level of subgenomic mRNA evidence, which would indicate active viral transcription. The debates continue (Krasemann, Glatzel, & Pless, 2022a; Vavougios et al., 2022) and it has been proposed that in mice and in vitro models, not the whole virus but just the S protein (or its cleaved‐off part, S1) is able to cross the BBB (Buzhdygan et al., 2020; Rhea et al., 2021). S1 may bind ACE2 in brain neurons and elevate levels of angiotensin II, inducing microglial activation and leading to tissue damage, within the paraventricular nucleus in the brain (Rodriguez‐Perez et al., 2015). SARS‐CoV‐2 infection in the CNS increases the expression of proteases, cytokines, and clotting factors; interferes with the tight junction proteins located between the endothelial cells of the BBB; and upregulates leukocyte trafficking in this tissue (Erickson et al., 2021). The infection also leads to mitochondrial dysfunction and rewiring of multiple pathways of the host cells (Medini et al., 2021; Nagu et al., 2021; Scozzi et al., 2021). All these events are probably the underlying cause of increased inflammation, demyelination, and decreased oxygen saturation of neuronal tissue of some COVID‐19 patients, with a clear similarity to neuronal aging (Erickson et al., 2021; Nagu et al., 2021).

The deleterious effects of SARS‐CoV‐2 infection on mitochondria have been already described. For example, it increases the generation of mitochondrial reactive oxygen species (ROS), and the expression of genes associated with glycolysis‐associated enzymes (Violi et al., 2020). This leads to a rewiring of the infected cell metabolism from the mitochondrial oxidative phosphorylation (OXPHOS) towards the cytosolic glycolysis. Increased expression of the pentose phosphate pathway (which is typically in equilibrium with glycolysis, and thus, elevated when glycolysis is increased) allows the virus to elevate production of nucleotides for replication (Icard et al., 2021). This rewiring triggered by the virus will ultimately contribute to a lower availability of ATP (Medini et al., 2021), leading to higher levels of ROS and oxidative damage in the host cell (Mohiuddin & Kasahara, 2021), further increasing cell death. Dysregulated bioenergetics has also been observed in cellular aging (Baltanas et al., 2013; Traxler et al., 2022). Infection by RNA viruses can lead to increased generation of ROS and their release into the cytoplasm, which supports the observation that SARS‐CoV‐2 infection is accompanied by elevated ROS, a typical consequence of OXPHOS dysregulation (Gatti et al., 2020; Li et al., 2021; Schwarz, 1996).

Cytochrome B (MT‐CYB), encoded in mitochondrial DNA (mtDNA), is a component of complex III of the Electron Transfer Chain (ETC). SARS‐CoV‐2 infection increases blood levels of circulating mtDNA and its fragments coding for MT‐CYB in patients hospitalized with COVID‐19, as well as a close relationship between these levels and the rate of fatalities or increased risk of intensive care unit admission (Scozzi et al., 2021). It is well known that mutations in mtDNA can contribute to the etiopathology and evolution of the main neurodegeneration (Maynard et al., 2015). These mutations can also deleteriously affect mitochondrial dynamics and mitophagy. In the context of the SARS‐CoV‐2 infection, ORF9b appears to facilitate the degradation of Drp1, deleteriously affecting the elongation of mitochondria and likely the entire fusion/fission balance (Ganji & Reddy, 2020; Shi et al., 2014; Srinivasan et al., 2021). Impaired mitophagy has been also described in SARS‐CoV‐2 infection (Shang et al., 2021). Damaged mtDNA, dysregulated fission and mitophagy have been broadly described in neuronal aging, and in the main neurodegeneration (Angiulli et al., 2018; Guitart‐Mampel et al., 2022; Patro et al., 2021; Pickrell & Youle, 2015; Solesio et al., 2012; Solesio, Prime, et al., 2013a; Solesio, Saez‐Atienzar, et al., 2013b).

Elevated inflammation accompanies SARS‐CoV‐2 infection, especially in severe cases of the disease (Ji et al., 2020). Neuroinflammation is also a hallmark of many of the most common neurodegeneration and neuronal aging (Guzman‐Martinez et al., 2019; Sparkman & Johnson, 2008). Mitochondria are both involved in the regulation of inflammation and deleteriously affected by inflammation. For example, one of the main mitochondrial mechanisms of the host response to viral infection through the regulation of inflammation is exerted via activation of the mitochondrial antiviralsignaling protein (MAVS). Mitochondrial exposure to viral SARS‐CoV‐2 proteins, probably via the interaction of ORF9b with the poly (C)‐binding protein 2 (PCBP2) and AIP4 (a E3 ubiquitin protein ligase) (Shi et al., 2014; Srinivasan et al., 2021), decreases the production of type‐I interferons IFN‐I (Shi et al., 2014). Thus, ORF9b seems to suppress MAVS signaling pathway (Singh et al., 2020), and, as described below, also interacts with mitochondrial import receptor protein TOM70. Lastly, some authors have found that ORF10 may induce degradation of mitochondria (including MAVS) via mitophagy, by targeting MAVS and the INF‐I signaling pathway (Li et al., 2022). Yet others have questioned if ORF10 is a protein‐coding gene (Figure 1).

**FIGURE 1:**
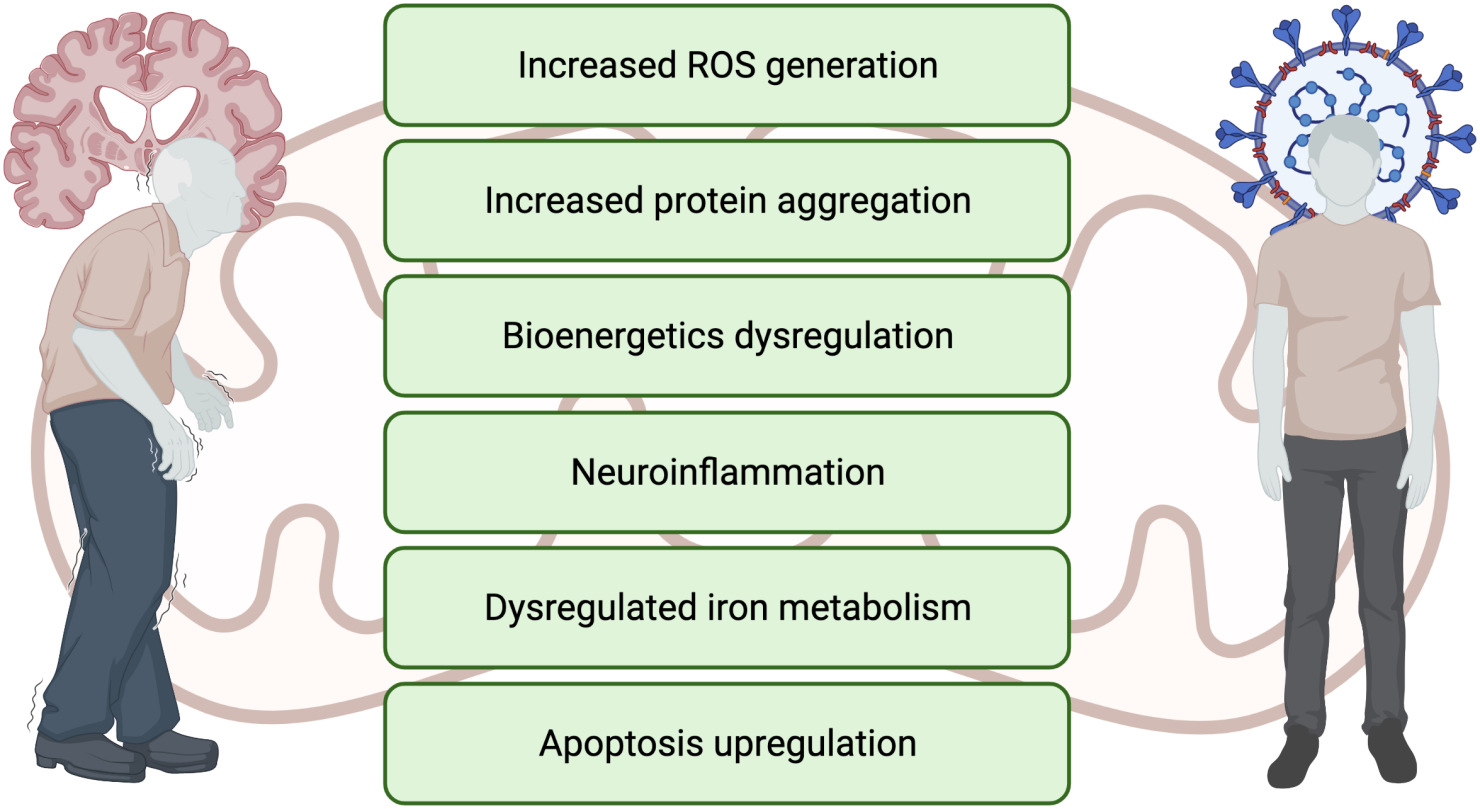
COVID‐19 and the main neurodegenerative disorders share some common mitochondrial disease mechanisms. Mitochondrial dysfunction is present in the etiopathology of all the main neurodegenerative disorders, as well as in COVID‐19. In fact, the effects of these two types of pathologies in the organelle are similar in many aspects.

SARS‐CoV‐2 can interact with the innate immune system using a mechanism involving mitochondrial regulation. Specifically, during viral infection, neutrophils produce and release ROS to degrade the invading virus (Laforge et al., 2020). Under pathological conditions, neutrophils may produce excess ROS, causing the local inflammation to become systemic (Mortaz et al., 2018), via a mechanism which involves neutrophil extracellular traps and apoptosis‐associated speck‐like proteins that contain a caspase recruitment domain. This process of local inflammation turning into systemic inflammation is common in severe COVID‐19 cases (Aymonnier et al., 2022), as demonstrated by a high neutrophil‐to‐lymphocyte ratio in these patients (Seyit et al., 2021). The activation of the inflammasomes, among other effects, further contributes to increased ROS generation, and hence, to mitochondrial dysfunction (Abais et al., 2015). Increased ROS is a crucial contributor towards the production of damage‐associated molecular patterns, integral to human innate immune response as they bind to intracellular receptors, contributing to the development of an adaptive immune response (Roh & Sohn, 2018).

Further involvement of mitochondria in the inflammatory response in COVID‐19 is suggested by the increased production of IL‐6 that can have broad consequences on mitochondrial dynamics. Specifically, it has been demonstrated that higher levels of IL6, a protein present in the SARS‐CoV‐2 infection and implicated in its severity (Patra et al., 2020), correlate with lower levels of TFAM, a protein that is involved in the regulation of the dynamics of the organelle, negatively affecting this process (Coomes & Haghbayan, 2020; Skuratovskaia et al., 2021). TFAM can bind to mtDNA and is essential for its maintenance (Kang et al., 2007; Skuratovskaia et al., 2021). SARS‐CoV‐2 also disrupts IFN‐I signaling via interfering with TOM70, a mitochondrial import receptor protein (Jiang et al., 2020). TOM70 has gained attention in the SARS‐CoV‐2 infection, as it has been demonstrated that ORF9b is associated to this protein, even if the studies show that this association is mainly with the cytosolic segment of the mitochondrial protein (Gao et al., 2021). Notably, in HEK293 cells the binding of ORF9b and TOM70 antagonized the innate immune activation, while TOM70 was required for the activation of MAVS (Thorne et al., 2022).

A crucial component of the inflammatory response closely related to the status of mitochondrial physiology is the activation of the hypoxia‐inducible factor 1α (HIF‐1α). HIF‐1α is an important activator in key pathways, such as glycolysis and OXPHOS (Clough et al., 2021; Kim et al., 2006); probably through its regulatory role in hypoxia and ROS generation (Codo et al., 2020; Semenza, 2011), or even via the regulation of the reverse electron transport (RET) in the ETC, a well‐known source of ROS (Clough et al., 2021; Scialo et al., 2017). Released in response to increased oxidative stress, even before the appearance of inflammation (Ke & Costa, 2006), HIF‐1α regulates the expression of many genes that are implicated in maintaining oxygen homeostasis as well as glucose uptake (Ziello et al., 2007). Within the CNS, recent studies have found increased production of ROS and HIF‐1α expression in microglia when exposed to SARS‐CoV‐2 (Clough et al., 2021). The elevated ROS production in these cells could lead to increased oxidative stress, further impacting immune function and inflammatory response, as well as increased mitochondrial dysfunction and cellular dyshomeostasis (Ruan et al., 2020; Wing et al., 2021). Hypoxia, which has been described in COVID‐19 patients and is the main cause of increased HIF‐1α (Serebrovska et al., 2020), may per se trigger mitochondrial dysregulation, acidosis, altered mitochondrial membrane permeability, and eventually, insufficiency of ATP biosynthesis; ultimately inducing cell death (Bargiela et al., 2018). It is important to note that some of these results seem to indicate a SARS‐CoV‐2‐specific mechanism, as when similar experiments were conducted with H1N1 (a subtype of influenza A) and respiratory syncytial virus, the changes in glycolysis were not observed (Codo et al., 2020).

Dysregulated apoptosis is present in the etiopathology of many human diseases, including the main neurodegeneration (Tait & Green, 2010; Xu et al., 2019), and in aging (Higami & Shimokawa, 2000). As previously mentioned, SARS‐CoV‐2 affects apoptosis in host cells, also within the CNS (Astuti & Ysrafil., 2020; Chan, 2020; Douaud et al., 2022; Ferreira et al., 2021; Iroegbu et al., 2020; Missiroli et al., 2020). [Correction added on 27 October 2022, after first online publication: Few incorrect reference citations in the above sentence were removed in this version]. In fact, increased apoptosis has been broadly described after SARS‐CoV‐2 infection, even if the exact mechanism that induce this effect still remains controversial and might be cell type‐dependent. For example, in HEK293 cells, it has been shown that SARS‐CoV‐2 increases apoptosis via the interaction of protein ORF7a of the virus with the Bcl‐X_L_ protein, a well‐known anti‐apoptotic protein (Tan et al., 2007). Further, in the case of the pulmonary edema which is found in some COVID‐19 patients, the viral M protein interacted with the Bcl‐2 and ovarian killer protein (BOK, a non‐canonical pro‐apoptotic member of the Bcl‐2 family) (Yang et al., 2022), mediating apoptosis. Other research groups have shown the importance of the ORF3a protein on the activation of apoptosis in several mammalian cell lines (Ren et al., 2020). ORF3a is a transmembrane protein that induces the activation of caspase‐8, which then cleaves Bid (a pro‐apoptotic protein (Billen et al., 2008)) to tBID and triggers the release of Cytochrome C.

The apoptotic effects of other SARS‐CoV‐2 proteins have also been investigated. The S protein of SARS‐CoV‐2 upregulated intracellular ROS generation in ACE2‐expressing human bronchial epithelial and microvascular endothelial cells (Li et al., 1867). The result of this rise in ROS generation is the suppression of the PI3K/AKT/mTOR signaling pathway, crucial in apoptosis signaling, and the consequent activation of autophagy, apoptosis, and inflammatory responses (Li et al., 1867). Increased ROS could also deleteriously directly impact mitochondrial physiology, further increasing apoptosis. ORF7b, an accessory protein of SARS‐CoV‐2 also affected the expression of several inflammatory cytokines, such as TNFα, IL‐6, and interferon β (IFN‐β) (Yang et al., 2021). Interestingly, under pathological conditions, TNFα is detected in other cells, such as neurons, where it is involved in the activation of the caspase cascade (Badiola et al., 2009).

As mentioned, there are some common cellular features of SARS‐CoV‐2 on the CNS and those of neurodegeneration (especially age‐related), including the presence of mitochondrial dysfunction. Increased dementia is seen in COVID‐19 patients, even if the mechanism(s) explaining this observation remain poorly understood. Some authors have proposed that this could be mediated by the overlapping neuroinflammation and microvascular injury present in both AD and COVID‐19, maybe also involving the dysregulation of the antiviral defense genes (Zhou et al., 2021). A recent study reported a rapid (ten days) worsening of the symptoms of Parkinson's Disease (PD) after SARS‐CoV‐2 infection, which ultimately led to an increased rate of death (Hainque & Grabli, 2020). This suggests that the SARS‐CoV‐2 infection can also affect motor abilities of patients, and even some nonmotor symptoms also worsened in PD patients infected by the virus (Brown et al., 2020). However, the low numbers of patients included in these studies have limited the statistical power of the findings and complicated the interpretation and extrapolation of the results.

In Genome‐Wide Association Studies, several immunity‐ and inflammatory‐linked genes have been associated with Alzheimer's Disease (AD) *(*Tosto & Reitz, 2013 *)*, and regulation of some of them changes in response to SARS‐CoV‐2 (Wang et al., 2022). For example, clusterin, also known as ApoJ, is one of the main apolipoproteins and an important chaperone in the human brain, where it has several functions. These include the regulation of diverse pro‐inflammatory cytokines, transport of lipids throughout the brain; and possibly its participation in the clearance of the amyloid β (Aβ) (Foster et al., 2019). Apart from AD, clusterin is also involved in the etiopathology of other major neurodegenerative disorders, such as amyotrophic lateral sclerosis, multiple sclerosis, and Huntington's disease (Foster et al., 2019). Its expression is promoted by stress‐related transcription factors (Foster et al., 2019) and it inhibits overall apoptosis (Zhang et al., 2005). This mechanism is negatively affected in neurodegeneration, where clusterin seems to be elevated (Lidström et al., 1998).

Interestingly, similar effects have been found in COVID‐19 patients (Singh et al., 2021).

While Aβ amyloids are characteristically present in the brain of AD patients, they also seem to be present in the cortexes of COVID‐19 patients. In a recent pre‐print publication, the authors showed increased presence of aggregated Aβ (and of hyperphosphorylated Tau, p‐Tau, another crucial amyloid in AD (Busche & Hyman, 2020)) in brains of both autistic patients and individuals with no underlying neuropsychiatric conditions (Shen et al., 2022). The authors stated that these aggregates were not found in age‐matched brains from both autistic and neurotypical individuals, who were tested negative for COVID‐19. While the number of individuals included in this study was again rather low, the authors conducted further studies using cellular samples to test their hypothesis. Specifically, they infected neurons derived from stem cells with SARS‐CoV‐2 virus and found that the deposition of Aβ and p‐Tau aggregates increased. Their data also suggested higher levels of apoptosis in these infected samples, evident by a rise in levels of caspase‐3 (Shen et al., 2022).

In a study conducted using 3D human brain organoids, increased presence and redistribution of p‐Tau in cortical neurons was found in SARS‐CoV‐2 samples compared with the controls (Ramani et al., 2020). The authors reported that even if the virus seems to not to actively proliferate in neurons, its presence in the CNS elevates cell death rates. Corroborating these findings, increased p‐Tau (similar to that found in AD) was found after infection with SARS‐CoV‐2 (Reiken et al., 2022). This study used human tissues obtained from hearts, lungs, and brains of COVID‐19 patients and control group, and reported elevated oxidative stress, as well as dysregulated calcium‐handling proteins in patients compared with controls. However, the levels of the amyloid precursor protein (APP) were not affected in these samples. The authors proposed that the mechanism explaining the observed effects could involve the presence of leaky ryanodine receptor 2 (RyR2), after SARS‐CoV‐2 infection. RyR channels are closely related to calcium homeostasis in the human brain (Datta et al., 2021; Meinhardt et al., 2021). Mitochondria are a crucial regulator of calcium levels in mammalian cells. These levels are closely related to the status of bioenergetics and the presence of oxidative stress, as we and others have demonstrated (Borden et al., 2021; Solesio et al., 2020; Solesio, Demirkhanyan, et al., 2016a; Solesio, Elustondo, et al., 2016b). In agreement with these findings, using HCN‐2 cells (a pediatric human cerebral cortical cell line) and transcriptomic techniques, some authors have shown a dysregulation of the antioxidant response, after SARS‐CoV‐2 infection, in HCN‐2 cells. They also linked the effects of SARS‐CoV‐2 infection and the induction of senescence (Valeri et al., 2021).

The presented studies have clear limitations. For example, those conducted in humans do not state the individuals' vaccination status, which may have an important impact on the viral load of the patients. In the studies conducted on animal and/or cellular models, for obvious reasons, the effects of the vaccine on the observed outputs cannot be evaluated. Additionally, pharmacological treatments against psychiatric or other symptoms may have an effect on certain infection routes, but they are not considered in many of the studies on patients. Lastly, increasing the sample size of the clinical studies will help to set the matter on a more reliable statistical footing.

## AUTHOR CONTRIBUTIONS

Writing this review was the central objective of a graduate level Rutgers University course (taught by MES) during the Spring semester of 2022. All the students listed as authors in this manuscript contributed equally to this work during the semester. However, the students listed as first authors, voluntarily extended their work into the summer semester, participating in the final writing and review before submission. Several students also participated in the Coronavirus course (taught by AG) at Rutgers University during the Spring semesters of 2021 and 2022. In that course, the current aspects of SARS‐CoV‐2 genomics and lifecycle, infection, and disease were discussed. RTDC is a postdoctoral researcher at Solesio Laboratory, who contributed to the review after the semester was over. AG contributed to the final writing and reviewed the manuscript before submission. MES contributed to the selection of the manuscripts that the students discussed, structured the review, coordinated, and led the writing process, and reviewed the manuscript before submission.

## CONFLICT OF INTEREST

The authors declare no conflict of interest.

## Data Availability

My article does not require one (this is a review) ;N/A
